# The Effect of Stimulus Area on Global Motion Thresholds in Children and Adults

**DOI:** 10.3390/vision3010010

**Published:** 2019-03-14

**Authors:** Kimberly Meier, Deborah Giaschi

**Affiliations:** 1 Department of Psychology, University of Washington, Guthrie Hall Box 351525, Seattle, WA 98105, USA; 2 Department of Ophthalmology and Visual Sciences, University of British Columbia, Rm E300E, 4480 Oak Street, Vancouver, BC V6H 3V4, Canada

**Keywords:** visual development, global motion, motion perception, speed

## Abstract

Performance on random-dot global motion tasks may reach adult-like levels before 4 or as late as 16 years of age, depending on the specific parameters used to create the stimuli. Later maturation has been found for slower speeds, smaller spatial displacements, and sparser dot arrays. This protracted development on global motion tasks may depend on limitations specific to spatial aspects of a motion stimulus rather than to motion mechanisms per se. The current study investigated the impact of varying stimulus area (9, 36, and 81 deg^2^) on the global motion coherence thresholds of children 4–6 years old and adults for three signal dot displacements (∆*x* = 1, 5, and 30 arcmin). We aimed to determine whether children could achieve mature performance for the smallest displacements, a condition previously found to show late maturation, when a larger stimulus area was used. Coherence thresholds were higher in children compared to adults in the 1 and 5 arcmin displacement conditions, as reported previously, and this did not change as a function of stimulus area. However, both children and adults performed better with a larger stimulus area in the 30 arcmin displacement condition only. This suggests that immature spatial integration, as measured by stimulus area, cannot account for immaturities in global motion perception.

## 1. Introduction

The study of the development of motion perception has revealed that, as with many visual functions, different aspects take longer to mature than others. For global motion, there are many parameters in the stimulus that can produce a change in sensitivity when varied. When changes in parameters differentially impact performance in children and adults, this is an important indicator of the trajectory of the developing motion system. Motion speed is one such parameter. For example, adult-like performance is reached sooner in life for global motion perception tasks using fast speeds, compared to slow speeds (random dot motion: [1,2]; Gabor patch motion: [3,4]). The speed of a global motion stimulus depends on the underlying spatiotemporal parameters (∆*x*/∆*t*) used to generate an animation on a computer screen. Studies of developing macaques [5,6] and humans [7,8] have demonstrated greater sensitivity early in life for motion created with large spatial displacements (∆*x*), compared to small spatial displacements, regardless of the temporal displacement (∆*t*). In a large cross-sectional study of observers between 7 and 30 years of age [8], we previously determined that adult-like performance was reached before age 7 for a large displacement (30 arcmin), around age 11 for a medium displacement (5 arcmin), and as late as age 16 for a small displacement (1 arcmin). This was the case for stimuli with a ∆*t* parameter of 17 ms (corresponding to speeds of 30, 5, and 1 deg/s, respectively) and of 50 ms (corresponding to speeds of 10, 1.7, and 0.3 deg/s, respectively). Along with evidence that performance in children with amblyopia is impaired at small and medium, but not large, displacements [9], these findings implicate a coarse-to-fine pattern of development in humans, such that sensitive periods for development are closed sooner in life for large spatial displacements, extending to the middle teenage years for stimuli with the smallest displacements, irrespective of stimulus speed.

The ability to integrate information from the multiple receptive fields that span the entire visual field is key not only in global motion perception, but in other aspects of spatial visual perception as well. Thus far, we have assumed that the immaturities in children revealed when manipulating the spatial displacement (or speed) of a motion stimulus reflect the function of a mechanism that operates at a global motion processing stage. However, if children show limitations in spatial integration at a stage prior to global motion integration, then the immaturities and deficits shown in the studies cited above may not solely be a function of motion processing mechanisms. Indeed, spatial integration tasks show similarly long developmental trajectories to those found for global motion perception: sensitivity for detecting Glass patterns becomes adult-like between the ages of 6–9 years [10], and contour detection thresholds improve until at least age 14 [11,12]. In developing macaques, Glass pattern detection [5] and contour integration [13] mature between 2–3 years of age (equivalent to 8–12 human years). This evidence highlights the possibility that spatial factors may underlie the apparent immaturities in motion perception observed in children. Thus, it is important to consider how manipulations of spatial aspects of motion stimuli may impact performance. This will allow us to rule out (or confirm) limitations at visual processing stages prior to global motion processing.

Dot density is one parameter that has been considered. Barlow and Tripathy [14] assessed performance in adults for two-frame dot motion stimuli using densities from 1.7 to 111 dots/deg^2^ and found only a very small improvement in direction discrimination coherence thresholds with increases in density. Global motion coherence thresholds in adults appear invariant to changes in density for a range of 1 to 30 [2] or 1.5 to 12.2 [15] dots/deg^2^. However, these studies also found that typically-developing children [2] and adults diagnosed with dyslexia, a developmental disorder that can also disrupt motion perception [15], perform better when presented with more dense displays, when all other stimulus parameters are held constant. Thus, global motion immaturities and deficits may be exaggerated when assessments are made with very sparse displays, which are more detrimental for the performance of children and observers with visual dysfunction, possibly due to immature spatial integration mechanisms [2]. Very dense dot displays, on the other hand, may yield mature performance. This possibility is consistent with adult-like global motion performance in 3–4 year old children observed in a study by Parrish, Giaschi, Boden, and Dougherty [16]: in addition to large values of ∆*x*, these stimuli were presented with a relatively high density of 32 dots/deg^2^.

Another parameter to consider is the area covered by a motion stimulus. Increasing area should lead to increasing sensitivity on a global motion task until the size of the stimulus reaches the size of the spatial summation area over which motion information is integrated, at which point sensitivity will plateau (e.g., [17]). In our previous work [7,8] we used stimuli that were square patches measuring 7.7 × 7.7 deg, for a total area covering 59.3 deg^2^. In healthy adults, both motion detection and direction discrimination thresholds decrease logarithmically with increases in stimulus area between 0.25 to 16 deg^2^ [18]. Additionally, Barlow and Tripathy [14] noted a log-linear improvement in thresholds as stimulus area was increased from 3 to 12 deg^2^, with little to no improvement for areas larger than this up to 171 deg^2^. From this evidence, it seems unlikely that adult performance would improve with stimulus areas larger than those we used previously. However, it is possible that effects of speed or ∆*x* on coherence thresholds may interact with effects of stimulus area; these effects could be different for adults, who may show no change in performance, while children may show improved, more adult-like performance with larger areas. If so, the developmental differences observed selectively for slow speeds or small ∆*x* in our previous studies [7,8] may be abolished if children are presented with larger stimulus areas.

Prior work on the impact of stimulus area in motion perception has assessed the smallest and largest ∆*x* displacement in a two-frame animation that is perceived as motion. Minimum ∆*x* displacement thresholds (*D_min_*) for direction discrimination in grating stimuli are unaffected by stimulus area unless the stimuli are presented in the periphery [19], suggesting that perception of global motion using small ∆*x* displacements may be unaffected by stimulus area. However, it has been established that maximum ∆*x* displacement thresholds (*D_max_*) can increase as stimulus area increases [20,21,22], and as retinal eccentricity increases [23]. This finding indicates that larger stimulus areas that cover more of the periphery may shift to or recruit additional motion mechanisms tuned to large or coarse displacements, and in turn, faster speeds. Psychophysical estimates of the size of receptive fields for motion detection using drifting gratings indicate larger receptive fields for stimuli with lower spatial frequencies [24,25] and electrophysiological MT responses match psychophysical responses well at faster speeds [26]. Taken together, these previous findings suggest that healthy adults will show better performance with increased stimulus area for large, but not small, ∆*x* displacements (for a constant ∆*t*). To our knowledge, no one has assessed the effect of area in children or in observers with disorders that impact visual development. Therefore, it is unknown if stimulus area has a differential effect on performance in these populations. Given that increases in stimulus density can lead to improved performance in children, it is important to investigate whether increases in stimulus area may have a similar effect.

The current study was designed to determine whether adults show differential effects of area for slow and fast stimuli; and, more importantly, whether manipulating stimulus area has the same impact on coherence thresholds in children between the ages of 4–6 years old. While we predicted that the performance of children will be immature for slow (∆*x* = 1 and 5 arcmin) but not fast (∆*x* = 30 arcmin) displacements based on our prior work with this age group [7], we were specifically interested in whether stimulus area and age have an interactive effect on coherence thresholds. Our aim was to determine if stimulus area could be discounted as a factor leading to the observed immaturities in performance by children on slow-speed or small ∆*x* motion tasks. If not, the previously-reported immaturities in global motion perception as a function of spatial displacement need to be re-considered.

## 2. Materials and Methods

This work was carried out in accordance with the Declaration of Helsinki and approved by the University of British Columbia’s Children’s and Women’s Research Ethics Board (ethical project code H12-03331).

### 2.1. Power Analyses

A power analysis was conducted using G*Power 3.1 [27] to determine the appropriate sample size for this study. Using estimates from our prior work comparing 4–6 year olds to adults [7], we calculated that 14 total participants (7 per age group) were required to replicate the main effect of age (Cohen’s *f* = 0.97) and 56 total participants (28 per age group) were required to replicate the age by ∆*x* interaction (*f* = 0.30) with a power of 0.80. Moreover, based on the magnitude of this interaction, we used *f* = 0.30 as the minimum meaningful effect size for detecting an age by stimulus area interaction in the current study. Thus, we determined we needed a total of 28 participants per age group for this experiment.

### 2.2. Participants

Children (4–6 years old) and young adults (18–30 years old) were recruited from the community to participate in this study. Participants had normal or corrected-to-normal vision and no self- or parental-reported visual, developmental, or cognitive disorders. No minimum visual acuity criteria were imposed, because prior work has shown that visual acuity does not impact coherence thresholds in participants with typically-developing visual systems [8], at least for the stimuli used here. However, participants with greater than 0.20 logMAR difference in visual acuity between their eyes were excluded from analysis, as this can be a risk factor for, or indicative of, amblyopia. Because motion processing and stereopsis share common cortical networks [28], participants were required to have stereoacuity scores within the normal limit for their age (200 arcsec for 4- and 5-year olds, 100 arcsec for 6-year olds, 40 arcsec for adults [29]).

A total of 33 children were recruited to participate. However, four children (ages 4.0, 4.8, 5.1, and 5.3) withdrew from the experiment after completing only two or three conditions, and one child (age 6.7) had poor stereoacuity; thus, their data were excluded from analysis. The remaining 28 children were between the ages of 4.2 and 6.9 years (*M* = 5.7, *SD* = 0.7; *n* = 4 four-year-olds; *n* = 15 five-year-olds; *n* = 9 six-year-olds). A total of 29 adults were recruited. One adult was excluded for having an acuity difference between their eyes that was 0.26 logMAR. The remaining adults included in the analyses were between the ages of 18.6 and 29.6 years (*M* = 23.2, *SD* = 3.5). No adults were excluded for poor stereoacuity.

### 2.3. Apparatus

Stimuli were generated with an Intel Core i7 Macintosh MacBook Pro running MATLAB R2015a (The MathWorks, Inc., Natick, MA, USA) equipped with the Psychophysics Toolbox extension version 3.0.12 [30,31,32]. Stimuli were presented on a BenQ XL2420T LED-backlit LCD monitor at a resolution of 1920 × 1080 and a 60 Hz refresh rate. Participants were seated in a dimly-lit room at a viewing distance of 1 m from the monitor, and responses were collected using a Gravis Gamepad Pro controller.

### 2.4. Stimuli and Experimental Conditions

The stimuli used in this experiment have been described previously [7,8,9]. Stimuli consisted of an array of 64 white (260 cd/m^2^) dots, 1 arcmin diameter, on a black (0.7 cd/m^2^) background, at a density of 1.1 dots/deg^2^ in each frame (or 1.7% of area). Signal dots moved left or right. Dot movement was controlled using a white noise algorithm: each time the animation frame was updated (approximately every 16.7 ms, equivalent to 60 Hz), a proportion of the dots, equal to the coherence value, were selected at random to be signal dots. The remaining dots were re-plotted in random locations. Thus, signal dot lifetime was determined probabilistically, such that the probability of each signal dot disappearing on each frame update was equal to the stimulus coherence level for any given trial. Stimulus duration was 600 ms (36 animation frames total). Density over time was 66 dots/deg^2^/s.

Two stimulus factors were examined: signal dot displacement and stimulus area. As in our prior studies [8,9], this study assessed performance for three spatial displacements (∆*x* = 1, 5, and 30 arcmin), but used only one temporal displacement (∆*t* = 17 ms). This combination of parameters yielded three speeds: slow (1 deg/s), medium (5 deg/s), and fast (30 deg/s). Three stimulus areas were assessed: 3 × 3, 6 × 6, and 9 × 9 deg squares; for total areas of 9, 36, and 81 deg^2^. Thus, a total of nine conditions were assessed. Density was held constant; thus, larger stimulus areas contained a greater number of dot elements.

### 2.5. Procedure

Prior to beginning the experiment, visual acuity was assessed using the Regan high-contrast letter chart [33] and stereoacuity was assessed using the Randot Preschool Stereoacuity Test (Stereo Optical Co., Inc., Chicago, IL, USA). Eight children who could not reliably name letters were assessed with the Lea Symbols picture chart [34].

**Figure 1:**
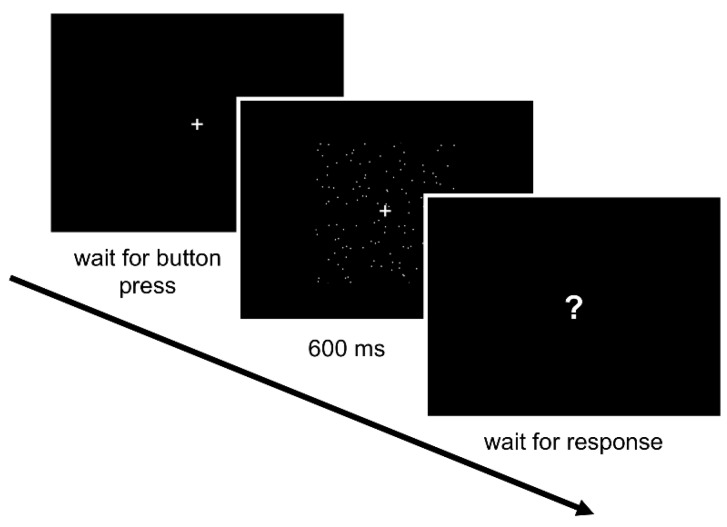
Schematic of the experimental procedure.

The experimental task was introduced to children and adults as a space-themed game in which the participant had to determine the direction of moving stars, left or right. To aid children with direction discrimination, images of a cartoon astronaut and cowboy were placed to the left and right of the monitor, respectively. Each trial began with a fixation cross in the center of the screen (Figure 1). After a button press, the motion stimulus was presented for 600 ms while the fixation cross remained on screen. When the stimulus finished playing, it disappeared and the fixation cross was changed to a question mark. Participants indicated their response using the controller (adults), or by pointing to the left or right side of the monitor or naming the cartoon character corresponding to the perceived direction of motion (children). Feedback was presented as an image of a particular cartoon character for correct responses, and a different cartoon character for incorrect responses. Finally, the fixation cross re-appeared in the center of the screen to initiate the next trial.

Performance was assessed using a hybrid approach [35,36], in which stimulus coherence levels were presented according to a staircase procedure and thresholds were calculated by fitting a psychometric function to the coherence by accuracy data. For each condition, stimulus coherence began at 100%. Trial-by-trial coherence was determined following a two-down one-up rule, such that coherence was decreased after two correct responses, or increased after one incorrect response. Coherence was changed in steps of 10% for the first three of these response reversals, after which the step size was halved at each reversal until a minimum step of 1% was reached. Reversals at coherence levels greater than 80% were not included in the rules for changing step size in case participants made an early mistake that prevented the staircase procedure from reaching a participant’s threshold. A consequence of this is that the estimate of coherence threshold for participants whose performance hovered around 80–90% coherence during the majority of the staircase run for a difficult stimulus condition (∆*x* = 30 arcmin, area = 9 deg^2^; see Results) is less precise due to the larger coherence step size. Staircases terminated after 50 trials or 10 response reversals, whichever occurred first.

Participants completed eight trials of a practice staircase binocularly using the parameters ∆*x* = 15 arcmin and ∆*t* = 33 ms at a stimulus area of 8 × 8 (64 deg^2^), before proceeding to the experimental conditions. Participants conducted the task monocularly using the eye with best visual acuity; if acuity between the eyes was equal, participants chose which eye to use. The order of conditions for each participant was determined using a Latin square.

### 2.6. Data Analysis

Coherence thresholds were calculated by fitting a Weibull function [37] to each participant’s responses using a maximum-likelihood parametric bootstrap procedure implemented in the Palamedes Toolbox [38]. The slope (β) of the function was free to vary; initial guess value was set to 3.5. Guess rate (γ) and lapse rate (δ) were fixed to 0.5 and 0.01, respectively, so that thresholds (α) corresponded to the coherence level for 81% correct performance. A Monte-Carlo goodness-of-fit test [39] implemented in the Palamedes Toolbox was used to assess the psychometric function fit for each threshold. Where this goodness-of-fit test failed, data were inspected and re-fit after removing an early mistake at high coherence levels and/or a trial reflecting a coherence level that was presented only once. If the fit could not be improved by this method, the participant’s threshold for this condition was removed from analysis. A total of 22 children and 25 adults had a complete set of data from all nine conditions. One child had time to complete only six of the nine conditions, and the remaining incomplete sets were due to poor staircase fits: one child had complete data for seven of the nine conditions, and four children and three adults had complete data for eight of the nine conditions.

**Figure 2:**
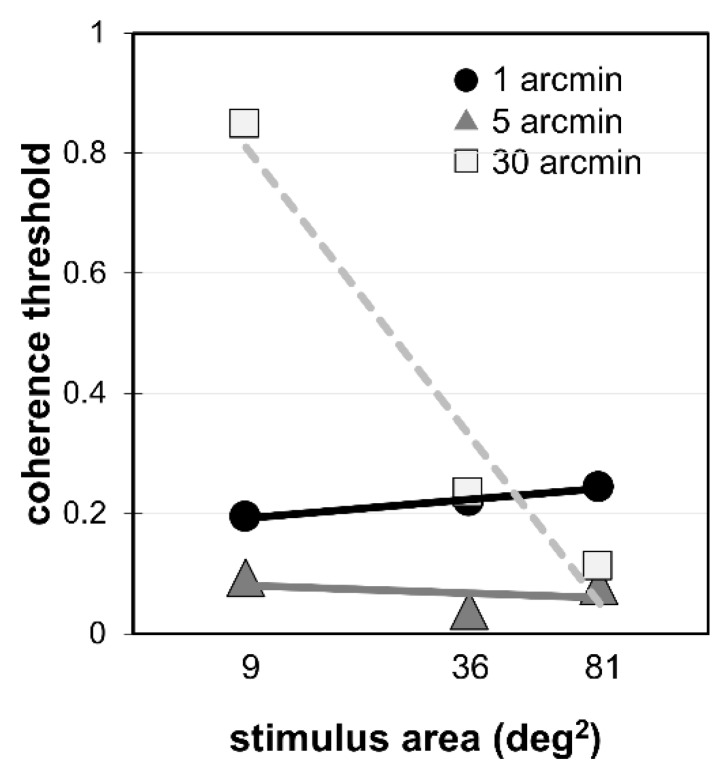
Effect of area for each ∆*x* for one adult participant. For this participant, the slope (text Section 2.6) is 0.05 for 1 arcmin; −0.02 for 5 arcmin; and −0.80 for 30 arcmin.

To quantify the effect of area, we obtained the slope of coherence thresholds as a function of the logarithm of the stimulus area for each participant. A negative value indicates that performance is better for larger stimulus areas. Figure 2 illustrates how these values were obtained for one participant. This slope value was used as a dependent variable in a subsequent analysis of variance using the between-subjects factor age (child, adult) and the within-subjects factor ∆*x* (1, 5, and 30 arcmin). Degrees of freedom were corrected with a Huynh-Feldt adjustment where Mauchly’s test indicated the assumption of sphericity had been violated (α = 0.25).

The benefit of expressing the effect of area with a slope, rather than entering area as the third factor in the ANOVA, is two-fold. For practical reasons, an estimate of the area effect can still be obtained for participants who do not complete all three ∆*x* conditions for a given area; thus, the analysis does not need to account for missing data. More importantly, quantifying the area effect as a slope provides a directly interpretable value. This value can also be compared with other studies.

## 3. Results

In adults, mean visual acuity for the eye used to conduct the task was −0.112 logMAR (approximately 20/15 Snellen; ranging from −0.213 to −0.025, *SD* = 0.058). In children, mean visual acuity was 0.058 logMAR (approximately 20/23 Snellen; ranging from −0.075 to 0.288, *SD* = 0.100). This difference (−0.170 logMAR or 1.7 lines on an eye chart) was significant, *t*(54) = 7.62, *p* < 0.001, however, children’s visual acuity was still within the normal range.

**Figure 3:**
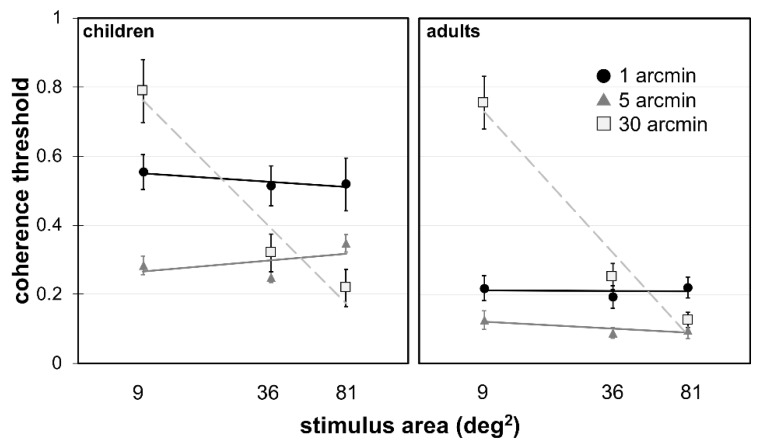
Coherence thresholds as a function of area for each ∆*x* condition, for the children (**left**) and adults (**right**). Error bars indicate 95% CI, determined individually around each mean. Linear fits describe the mean slope for each ∆*x* as a function of log-transformed stimulus area; confidence intervals for these slope estimates are displayed in Figure 4.

Mean coherence thresholds for each ∆*x* by area condition are shown separately for children and adults in Figure 3, along with the mean fitted slopes quantifying the area effect. To facilitate comparison with our previous studies, we conducted a two-way ANOVA on this data at the closest stimulus area to our previous studies, 36 deg^2^, for the participants who had a full dataset available (*n* = 26 children, 28 adults). There was a significant effect of ∆*x*, *F*(2, 104) = 31.51, *p* < 0.001, $\eta_{p}^{2}$ = 0.37; a significant effect of age group, *F*(1, 52) = 54.51, *p* < 0.001, $\eta_{p}^{2}$ = 0.51; and a significant ∆*x* by age group interaction, *F*(2, 105) = 16.22, *p* = 0.001, $\eta_{p}^{2}$ = 0.24. A simple main effects analysis found that children had significantly higher coherence thresholds for ∆*x* of 1 arcmin, *F*(1, 156) = 80.16, *p* < 0.001, *d* = 2.54; and 5 arcmin, *F*(1, 156) = 19.78, *p* < 0.001, *d* = 1.05; but not 30 arcmin, *F*(1, 156) = 3.54, *p* = 0.062, *d* = 0.55.

**Figure 4:**
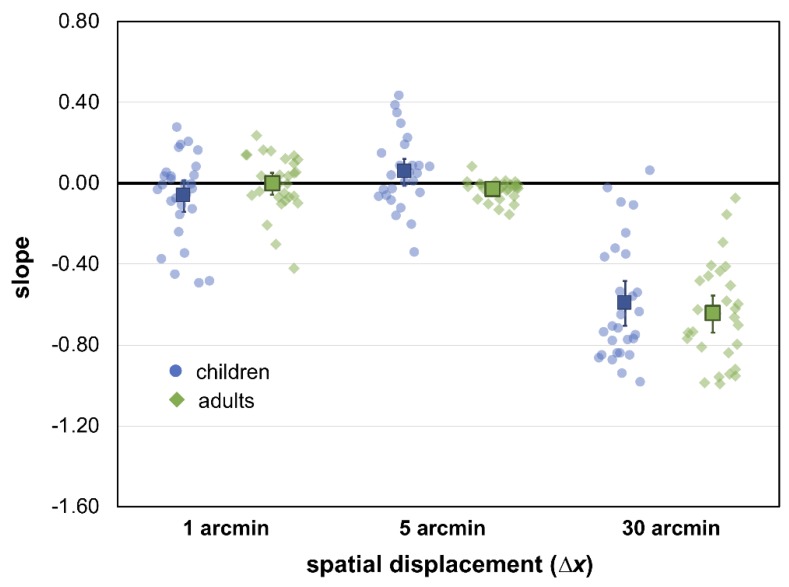
Slopes for each ∆*x* condition for both age groups. Error bars indicate 95% CI, determined individually around each mean. A value of 0 reflects no effect of area on coherence thresholds. A negative value indicates coherence thresholds are lower (improve) for larger stimulus areas.

The slope for each ∆*x* condition in each age group is re-plotted in Figure 4. There was a significant main effect of ∆*x*, *F*(1.6, 85.8) = 160.60, *p* < 0.001, $\eta_{p}^{2}$ = 0.75, but no significant effect of age group, *F*(1, 54) = 0.51, *p* = 0.48, $\eta_{p}^{2}$ = 0.01, nor a ∆*x* by age group interaction, *F*(1.6, 85.8) = 1.96, *p* = 0.15, $\eta_{p}^{2}$ = 0.03. Follow-up of the significant ∆*x* effect using Bonferroni-adjusted pairwise comparisons indicated that the slope for 30 arcmin was significantly greater than the slope at 1 and 5 arcmin (both *p* < 0.001), but the slopes at 1 and 5 arcmin were not different from each other (*p* = 0.76). The slope at 30 arcmin (*M* = −0.62, 95% CI: [−0.69, −0.55]) was significantly less than zero, *t*(55) = 16.99, *p* < 0.001. The slope at 1 arcmin (*M* = −0.03, 95% CI: [−0.08, 0.01]) and at 5 arcmin (*M* = 0.01, 95% CI: [−0.03, 0.05]), was not significantly different from zero, *t*(55) = 1.38 and 0.54, *p* = 0.17 and 0.61, respectively, indicating that performance did not differ as a function of stimulus size for these smaller ∆*x* displacements.

## 4. Discussion

We assessed coherence thresholds in young children (aged 4–6 years old) and adults as a function of stimulus area for three spatial displacements (∆*x* = 1, 5, and 30 arcmin). Previous work has indicated that young children show immature performance at the smallest, but not largest, ∆*x* values assessed in the current study [7,8]. The results of the current study replicate these prior results, and confirm that these immaturities are not limited by the spatial extent of stimulus area. Consistent with our hypothesis, we found an area effect at the fastest ∆*x* (30 arcmin) only. The effect of area was the same for children and adults, regardless of ∆*x* used in the stimulus.

This work assessed motion coherence thresholds as a function of speed and stimulus area, and found that thresholds vary as a function of area for large ∆*x* displacements only. This is consistent with prior work [40] that determined that the performance improvements in adults as stimulus area is increased from 8 to 18 deg^2^ do not differ for a 4 and 12 deg/s. Assuming that study used a constant ∆*t* locked to the refresh rate of the monitor (85 Hz or 11.8 ms), they used ∆*x* = 2.8 and 8.5 arcmin to create these speeds, which are closer to the small and medium displacements used in the current study. The current data are also consistent with prior work on the impact of stimulus area on *D_max_* in two-frame random dot kinematograms. Baker and Braddick [20] determined that *D_max_* varies strongly as a function of stimulus area: at the largest area assessed in the current study, 81 deg^2^, their upper displacement limit approached 40 arcmin, at our medium area of 36 deg^2^ their limit was around 30 arcmin, and at the smallest area assessed in the current study, 9 deg^2^, they found maximum displacement limits around 20 arcmin. Given that the slow, medium, and fast speeds in the current study were created using ∆*x* = 1, 5, and 30 arcmin, respectively, it is not surprising that no area effects were found for slow and medium speeds. In fact, the data from Baker & Braddick predict that an area effect would not be apparent for these speeds in the current study unless stimulus areas smaller than 1 deg^2^ were assessed.

The significant impact of stimulus area on stimuli with the large displacement used here (∆*x* = 30 arcmin) can be accounted for by limitations in *D_max_*. Global motion direction discrimination becomes difficult at our medium-sized area of 36 deg^2^, relative to the large stimulus area of 81 deg^2^, because the displacement used to move signal dots is equal to the two-frame *D_max_* of 30 arcmin reported by Baker and Braddick [20]. This is reflected by a mean increase of 11% for coherence thresholds between the 81 and 36 deg^2^ conditions in our participants (see Figure 3). Participants have even greater difficulty at the small stimulus area of 9 deg^2^, where 30 arcmin displacements are larger than the two-frame *D_max_* of 20 arcmin for this stimulus size reported in Baker and Braddick [20]. This is reflected in a mean increase of 49% in coherence thresholds between the 36 and 9 deg^2^ stimulus area conditions. Participants are still able to conduct the task, likely due to sequential recruitment mechanisms that increase *D_max_* by taking advantage of multiple animation frames [41]. It should be noted that the trajectory of a dot moving with a displacement of ∆*x* = 30 arcmin can only be updated a maximum of six times in the 9 deg^2^ area before reaching the edge of the stimulus, while it can be updated a maximum of 12 times in a 36 deg^2^ stimulus area, which may also contribute to the high coherence thresholds seen in the small area. Our data indicate that the decrease in coherence threshold is linear with logarithmic increases in stimulus area for the large displacement, which is consistent with previous literature assessing direction discrimination thresholds in global motion [14] and maximum displacement [20,42].

In this study, we found that although children show immature performance compared to adults for motion stimuli with small and medium (1 and 5 arcmin) but not large (30 arcmin) ∆*x* displacements, replicating prior work [7,8], this pattern of immaturity does not vary as a function of stimulus area. Previous research using an equivalent noise paradigm (presumed to be an integration task), rather than the motion coherence paradigm used here (presumed to be a segregation/integration task [43]), has suggested that children’s performance may be limited by an immaturity in sampling efficiency [1,44], that is, by the inability to make full use of the information available in a motion stimulus. If this is the case, increasing the area of the stimulus does not cause an increase in sampling efficiency that is greater for children than for adults. Instead, immaturities in children’s performance on motion direction-discrimination tasks likely occur from the motion energy available in more local motion signals. The work showing selectively improved performance in children [2] or people with dyslexia [15] relative to healthy adults when stimulus density is increased is consistent with this idea.

The results of the current study indicate that the immaturities observed at small ∆*x* described in previous studies [7,8] cannot be accounted for by limitations in spatial integration that selectively impact children’s performance, at least when spatial integration is indexed by stimulus area. Moreover, these results indicate that a difference in stimulus area is unlikely to underlie some of the inconsistent results reported across studies of global motion maturation reported in the literature.
